# The Onset of Falls Reduces Subjective Well-Being. Findings of a Nationally Representative Longitudinal Study

**DOI:** 10.3389/fpsyt.2021.599905

**Published:** 2021-03-30

**Authors:** André Hajek, Hans-Helmut König

**Affiliations:** Department of Health Economics and Health Services Research, University Medical Center Hamburg-Eppendorf, Hamburg, Germany

**Keywords:** fall, life satisfaction, positive affect, negative affect, SWLS, PANAS

## Abstract

**Introduction:** The prevalence of older individuals experiencing a fall is high. Moreover, falls can have deleterious effects on health status. Additionally, falls can affect the subjective well-being of individuals. However, there is a lack of studies examining the link between falls and subjective well-being. Therefore, the objective of this study was to investigate whether the onset of falling is associated with (intraindividual) decreases in subjective well-being in men and women.

**Materials and Methods:** Longitudinal data (from wave 5 to wave 6) were taken from a population-based sample of individuals residing in private households in Germany [in our analytical sample: 3,906 observations (men), and 3,718 observations (women)]. Positive and negative affect were quantified using the Positive and Negative Affect Schedule (PANAS). Life satisfaction was assessed using the Satisfaction with Life Scale (SWLS).

**Results:** Adjusting for various potential confounders, fixed effects regressions showed that the onset of falls was associated with a decrease in positive affect (β = 0.08, *p* < 0.01), and an increase in negative affect (β = 0.07, *p* < 0.01) among men. While the onset of falls was not associated with changes in positive affect in women, it was associated with a decrease in negative affect in women (β = 0.06, *p* < 0.05). Sex differences were significant. The onset of falls was not associated with decreases in life satisfaction in men, nor in women.

**Discussion:** The present study particularly highlights the link between the onset of falls and reduced affective well-being among men. Avoiding falls may contribute to maintaining affective well-being among middle-aged and older men.

## Introduction

Falls usually refer to “an unexpected event in which the participants come to rest on the ground, floor, or lower level” (1). The prevalence of older individuals experiencing a fall is high and increases with age (2). This is worth emphasizing since falls can cause morbidity and mortality (3). Furthermore, falls can cause an increased medical and financial burden (4).

Apart from these health-related and economic consequences, several studies have investigated the link between falls and psychosocial factors. For example, falls are associated with decreased autonomy (5), as well as increased loneliness and feelings of social isolation (6, 7). Studies have also shown that falls are associated with increased depressive symptoms or anxiety (8). Furthermore, falls are associated with a reduced *health-related* quality of life (9). However, only very few studies have focused on the link between falls and subjective well-being (SWB). It should be emphasized that SWB and health-related quality of life are different constructs (10). While SWB mainly refers to the way individuals feel and think about their lives, health-related quality of life mainly refers to health issues. SWB has two main components: While (i) affective well-being refers to the experience of negative (NA) and positive affects (PA), (ii) life satisfaction (also known as cognitive well-being) refers to the cognitive evaluation of life as a whole (11). Negative affect refers to negative emotions such as anger or distress and positive affect refers to positive emotions such as joy or excitement.

Based on a small cross-sectional study of 249 individuals in Hong Kong (China), Leung (12) showed that falls are associated with lower life satisfaction. In this study, life satisfaction was quantified using the 18-item version of the Life Satisfaction Index A (13). Furthermore, another cross-sectional study based on data from the German Aging Survey showed that falls were associated with lower life satisfaction in Germany (14). Moreover, falls were associated with higher negative affect and lower positive affect (14). However, to date, previous studies have not examined whether the onset of falling is associated with decreases in SWB. Only one longitudinal study of *n* = 1,321 individuals in a specific region in Sweden comprising of two waves (15) showed that fallers had a considerably lower life satisfaction score compared to non-fallers. However, this study did not focus on the onset of falling (i.e., intraindividual changes from non-falling to falling in the observation period). Therefore, our aim was to clarify whether the onset of falling is associated with decreases in subjective well-being stratified by sex, based on longitudinal data from a population-based study of middle-aged and older adults in Germany. Knowledge regarding this link is important, as SWB is a main goal for nations, due to its associations with various valuable societal outcomes (16, 17). SWB can contribute to health as well as longevity (18), and is also associated with successful aging (19).

Since falls are associated with various adverse health outcomes (as mentioned above), we hypothesize that the onset of falls is associated with decreases in life satisfaction in both women and men. Moreover, we hypothesize that the onset of falls is associated with decreases in positive affect in women and men. Additionally, we hypothesize that the onset of falls is associated with increases in negative affect in women and men. We hypothesize that decreases in subjective well-being are significantly more pronounced following the onset of falls in men (compared to women) because older men often report more frequent and severe falls (20–22).

## Materials and Methods

### Sample

In our study, longitudinal data from the German Aging Survey (“Deutscher Alterssurvey,” DEAS) were used. The DEAS is an ongoing longitudinal cohort-based survey. Community-dwelling older adults aged 40 and over were included in the DEAS study. Consequently, the main inclusion criterion was that individuals were 40 years or older. More precisely, inclusion criteria for first time participants were as follows: born between the years 1929 and 1974, as well as living in private households (and excluding individuals living in institutionalized settings). Inclusion criteria for panel participants were having one or more valid interviews, written consent to participate in the panel provided by baseline participants, as well as still being alive at the time of the panel study and not living abroad.

The DEAS started in 1996. The following waves were in 2002 (second wave), 2008 (third wave), 2011 (fourth wave), 2014 (fifth wave), and most recently in 2017 (sixth wave). The DEAS study has a cohort-sequential design. Cross-sectional samples were introduced in the second, third and fifth wave, while the fourth and the sixth wave only included individuals who had already taken part before (panel sample). In the most recent sixth wave, the response rate was ~63%. In the fifth and sixth wave, data were collected using computer-assisted personal interviews (CAPI). Furthermore, the individuals are asked to fill out an additional written questionnaire (covering more sensitive topics such as subjective well-being). The DEAS study is described in further detail elsewhere (23).

Because falls were only measured in the fifth wave with about 10,300 individuals and sixth wave with more than 6,600 individuals, we focused on these waves in our current study. In our analytical sample individuals were included who participated both in wave 5 and wave 6. When life satisfaction served as outcome measure, we included 3,906 observations (1,953 individuals; men) and 3,718 observations (1,859 individuals; women) in regression analysis.

Written informed consent was provided by all participants. The DEAS study follows the principles of the Declaration of Helsinki. As the criteria for an ethical statement were not fulfilled, such as risk for the respondents or use of invasive methods, an ethics committee approval was not required for the DEAS study.

### Dependent Variables

The German version of the Positive Affect and Negative Affect Schedule (PANAS) was used to assess PA and NA (24). Each of the scales (PA and NA) consists of 10 positive and negative feelings, respectively. The 10 negative feelings were distressed, upset, guilty, scared, hostile, irritable, ashamed, nervous, jittery, and afraid. The 10 positive feelings were enthusiastic, excited, strong, interested, proud, alert, inspired, determined, attentive, and active. Both scales range from one to five (higher values correspond to higher PA or NA, respectively). In our study, Cronbach's alpha was 0.87 for PA and 0.85 for NA. Watson, Clark and Tellegen (24) demonstrated very good psychometric characteristics of the PANAS. It should be noted that the German version has been validated (25).

Life satisfaction was quantified using the German version of the Satisfaction with Life Scale (SWLS) (26) consisting of five items. The final scale ranges from one to five (higher values correspond to higher satisfaction with life). Cronbach's alpha was 0.85 in our study. Pavot and Diener (26) demonstrated favorable psychometric properties of the SWLS. The German version has been validated (27).

### Independent Variables

A common way to measure falls history was used (28): The experience of a fall in the preceding 12 months (no or yes).

As covariates, we adjusted for several socioeconomic factors in the regression analysis: age, family status (married, living together with spouse; married, living separated from spouse; single; divorced; widowed), labor force participation (employed; retired; not employed), and household net equivalent income.

Furthermore, we adjusted for several health-related factors in the regression analysis: self-rated health (from 1 = very good to 5 = very bad), physical functioning [subscale “Physical functioning” of the SF-36 (29); from 0 (worst score) to 100 (best score)] and the number of physical illnesses (e.g., joint, bone, spinal, or back problems; ranging from 0 to 11).

### Statistical Analysis

Stratified by sex, linear fixed effects (FE) regressions were performed to analyze the link between falls and SWB [(1) life satisfaction, (2) PA, and (3) NA as outcome measures] longitudinally. This is, for example, in accordance with the recommendations given by Ferrer-i-Carbonell and Frijters (30). Various previous studies also used FE regressions to examine the determinants of SWB (31, 32). Ferrer-i-Carbonell and Frijters (30) showed that it is important to control for time-constant unobserved factors when estimating the determinants of SWB. In contrast to other panel regression techniques, the key advantage of FE estimates is that they are not biased by time-constant factors (both, observed and unobserved) that are systematically correlated with the explanatory variables—which is empirically often the case. A Hausman-test supported our choice to use FE regressions.

FE estimates solely rely on intraindividual changes within individuals over time (within-variation). In our study, we were interested in the link between the onset of falls and changes in SWB from wave 5 to wave 6. Since no within-variation in time-constant factors such as sex exists, these factors cannot be included as main effects in FE regressions. Hence, FE regressions were stratified by sex. In further FE regression analysis, we also tested whether the link between the onset of falls and changes in SWB significantly differs by sex by including respective interaction terms: sex × falls.

Cluster-robust standard errors were computed. Statistical analyses were performed using Stata 15.1. The level of statistical significance was set to 0.05.

## Results

### Sample Characteristics

Sample characteristics for individuals included in FE regression analysis (stratified by sex) are described in Table 1. Among the total sample, average age was 65.4 years (SD = 10.6 years; ranging from 40 to 97 years). Stratified by sex, average life satisfaction was 3.8 (SD = 0.7) in men, and 3.9 (SD = 0.7) in women. Further details are given in Table 1.

**Table 1:** Sample characteristics for individuals stratified by sex (men: *n* = 3,906 observations; women: *n* = 3,718 observations) included in linear fixed effects regressions (wave 5 to wave 6, pooled).

**Variables**	**Men**	**Women**
**Education (ISCED-97):** ***N*** **(%)**		
Low education	60 (1.5)	246 (6.6)
Medium education	1,748 (44.8)	2,050 (55.1)
High education	2,098 (53.7)	1,422 (38.3)
Age (in years): Mean (SD)	66.5 (10.7)	64.2 (10.5)
Married, living together with spouse: *N* (%)	3,049 (78.1)	2,347 (63.1)
**Employment status:** ***N*** **(%)**		
Employed	1,328 (34.0)	1,393 (37.5)
Retired	2,425 (62.1)	1,917 (51.5)
Other: not employed	153 (3.9)	408 (11.0)
Household net equivalent income (in Euro): Mean (SD)	2,153.6 (1,293.9)	1,974.1 (1.149.0)
Self-rated health (from 1 = very good to 5 = very bad): Mean (SD)	2.5 (0.8)	2.4 (0.8)
**Self-rated health:** ***N*** **(%)**		
Very good	315 (8.1)	325 (8.7)
Good	1,905 (48.8)	1,825 (49.1)
Average	1,330 (34.0)	1,267 (34.1)
Bad	303 (7.7)	260 (7.0)
Very bad	53 (1.4)	41 (1.1)
Physical functioning [from 0 (worst) to 100 (best)]: Mean (SD)	85.0 (20.1)	82.0 (22.3)
Number of physical illnesses (from 0 to 11): Mean (SD)	2.6 (1.9)	2.4 (1.9)
**Number of physical illnesses (from 0 to 11):** ***N*** **(%)**		
Absence of physical illnesses	438 (11.2)	455 (12.2)
Presence of 1 physical illness	840 (21.5)	868 (23.4)
Presence of 2 or more physical illnesses	2,628 (67.3)	2,395 (64.4)
Absence of falls: *N* (%)	3,424 (87.7)	3,044 (81.9)
Life satisfaction (from 1 to 4; higher values correspond to higher perceived life satisfaction): Mean (SD)	3.8 (0.7)	3.9 (0.7)

*Please note that sex and education were not included in FE regressions as explanatory variables because they do not vary within individuals over time (in this dataset). They are only depicted for descriptive purposes. Low education (ISCED = 0–2): respondents without formal vocational qualification); medium education (ISCED = 3–4: respondents with vocational training (at work or at school), including respondents with a higher general school certificate with-out professional training); high education (ISCED = 5–6: respondents with completed professional development training [professional, master or technical school, university of cooperative education, or academies] and respondents with completed university studies [university or university of applied science])*.

### Regression Analysis

We focused on the onset of falls i.e., individuals who did not experience a fall in the fifth wave and who reported a fall in the sixth wave. In total, 599 individuals reported such a transition (267 men; 332 women). It should be emphasized that FE regression estimates for the link between the onset of falls and SWB exclusively rely on these 599 individuals (267 men and 332 women). Because some individuals also had changes in other explanatory variables (e.g., self-rated health), they were also included in linear FE regressions (to investigate whether changes in these independent variables are associated with changes in SWB). For example, in sex-stratified FE regression analysis and with life satisfaction as outcome measure, 3,906 observations (1,953 individuals) were used (for men), while 3,718 observations (1,859 individuals) were used (for women).

Adjusting for numerous covariates, linear FE regressions revealed that the onset of falls was not associated with decreases in life satisfaction in men nor in women (see Table 2). However, in men, the onset of falls was associated with a decrease in PA (β = 0.08, *p* < 0.01), as well as an increase in NA (β = 0.07, *p* < 0.01). In women, the onset of falls was not associated with changes in PA, whereas the onset of falls was associated with a decrease in NA (β = 0.06, *p* < 0.05). Sex differences were significant (falls × sex, with PA as outcome measure: β = 0.10, *p* < 0.01; falls × sex, with NA as outcome measure: β = −0.13, *p* < 0.001).

**Table 2:** Longitudinal association between onset of falls and subjective well-being.

**Independent variables**	**Life satisfaction—Men**	**Life satisfaction—Women**	**Positive affect—Men**	**Positive affect—Women**	**Negative affect—Men**	**Negative affect—Women**
Onset of falls (Ref.: Not experiencing a fall)	−0.03 (0.04)	0.01 (0.04)	−0.08 (0.03)^**^	0.02 (0.03)	0.07 (0.03)^**^	−0.06^*^ (0.03)
Potential confounders	✓	✓	✓	✓	✓	✓
Constant	3.39 (0.31)^***^	4.23 (0.34)^***^	3.06 (0.23)^***^	3.20 (0.23)^***^	2.14 (0.23)^***^	2.20 (0.23)^***^
Observations	3,906	3,718	3,908	3,722	3,906	3,722
Number of Individuals	1,953	1,859	1,954	1,861	1,953	1,861
*R*^2^	0.02	0.02	0.03	0.03	0.01	0.04

*Results of linear FE regression analysis (wave 5 and wave 6)*.

Potential confounders include age, family status, labor force participation, self-rated health, income, physical functioning, and number of physical illnesses; beta-coefficients are reported; cluster-robust standard errors in parentheses;

*** *p < 0.001*,

** *p < 0.01*,

* *p < 0.05, +p < 0.10*.

## Discussion

### Main Findings

Based on longitudinal data from a nationally representative sample, the objective of this study was to investigate whether the onset of falling is associated with decreases in SWB (stratified by sex). We hypothesized that the onset of falls is associated with decreases in subjective well-being in both women and men (with more pronounced effects in men). However, our hypotheses were only partly confirmed: Adjusting for various potential confounders, FE regressions showed that the onset of falls was associated with a decrease in PA, and an increase in NA among men. While the onset of falls was not associated with changes in PA in women, it was associated with a decrease in NA in women. Sex differences were significant. The onset of falls was not associated with decreases in life satisfaction in men, nor in women.

To date, only three studies have examined the link between falls and life satisfaction (14, 15, 33). Two of these studies were cross-sectional (14, 33), and one study (15) was longitudinal. They also showed an association between falls and decreased life satisfaction (14, 15, 33). In contrast, in our study regressions showed no association between the onset of falls and a decrease in life satisfaction. We assume that these differences may be mainly explained by the large differences in statistical analysis (i.e., using cross-sectional regression techniques vs. using regression models for panel data) and the time horizon (34). More specifically, it is well-known that estimates derived from cross-sectional regression models can differ largely from estimates derived from panel regression models [e.g., (30)]. Furthermore and with regard to the time horizon, similar to the effect of other life events on SWB (35) it appears to be plausible that the onset of a fall only has a short-term effect—e.g., for a few months—on life satisfaction. In the mid-term and in accordance with previous research examining the impact of life events on well-being (36, 37), life satisfaction may return to its set-point because life satisfaction refers to the cognitive evaluation as a whole and is therefore mainly retrospectively oriented. Consequently, these individuals may quickly adapt to the consequences of falls. This may be supported by a study conducted by Powdthavee (38) based on longitudinal data from the British Household Panel Survey. He showed that the life satisfaction returns to his set point already after 1 year when a mild disability—a critical life event that both can have social and health consequences—occurs. Nevertheless, future longitudinal studies (with short time intervals) are required to shed further light onto this issue.

Only one cross-sectional study examined the association between falls and affective well-being (14). Our longitudinal study extends this knowledge by showing that the onset of falls is associated with decreases in affective well-being in men. It is puzzling that differences exist between men and women in the way the onset of falls is associated with affective well-being. One explanation may be that men may experience more severe and more frequent falls (e.g., due to more demanding past and current physical activities) (20–22). We assume that the onset of falls may have adverse health consequences for both women and men. It may be the case that these impairments differentially affect men and women. More specifically, a possible, but speculative, explanation may be that men have more difficulties in coping with the consequences of falls, for example in terms of fear of falling or avoidance of physical activities. A previous qualitative study supports this idea (39). Possibly, they might not want to restructure their more physical-related goals. This might ultimately contribute to a decreased affective well-being in men. However, while studies exist focusing on the moderating role of flexible goal adjustment or tenacious goal pursuit on the link between health-related independent variables and well-being outcomes (40, 41), these previous studies did not focus on the role of sex differences in this link. Hence, future studies including three-way-interactions (falls × sex × flexible goal adjustment or tenacious goal pursuit) are required to verify our assumptions.

In contrast, women may find it easier to restructure their goals and focus more on cognitive or social activities. Therefore, they may be able to maintain their positive affect—or even decrease their negative affect. However, the latter association is surprising. Overall, it is unexpected that the onset of falls is associated with a *decrease* in NA among women in our study. We also ran further robustness checks (by adding loneliness, social isolation, autonomy, self-esteem, and depressive symptoms to our main model). However, the association between the onset of falls and a decrease in NA in women remained almost the same (results not shown, but available upon request). We also checked whether this link varies by age group (individuals 40–64 years; 65 years and above) or by the educational level. However, the association remained virtually the same (results not shown, but available upon request). Thus, we assume that is not a spurious effect. In line with the argument above, a highly speculative explanation may be that women who experienced a fall may subsequently receive more assistance from family or friends. This increase in social support may be perceived as an enrichment. However, there is a lack of studies supporting our speculative, possible explanations. Thus, future research is required to verify this speculative assumption. Others may argue that this increase in social support caused by a fall may be perceived as burden from individuals who experienced a fall.

### Strengths and Limitations

Some strengths are worth noting. Longitudinal data were taken from a population-based study of community-dwelling individuals aged 40 and over. We provide first insights into the longitudinal association between the onset of falls and SWB. SWB was measured using widely used and well-validated scales (SWLS and PANAS). We adjusted for several covariates such as age, marital status, employment status, self-rated health, physical functioning, or physical illnesses in regression analysis. However, some important covariates may be missing such as personality factors (42). Some further limitations are worth acknowledging. Women, less educated individuals, individuals living in large cities and individuals aged 70–85 years have a lower probability of participating in the DEAS study. This means that a small sample selection bias is present in the DEAS study (43). Self-rated falls in the last 12 months were used in our study as the outcome measure. Therefore, the possibility of a recall bias cannot be entirely dismissed. However, it is plausible that individuals recall a fall in the preceding year (15). Future research is required to clarify whether the severity and the number of falls is differently associated with SWB longitudinally. Moreover, more complex models such as dynamic panel-data estimation using maximum likelihood and structural equation modeling could be used in upcoming studies (44).

## Conclusion

The aim of this study was to investigate whether the onset of falling is associated with (intraindividual) decreases in subjective well-being in men and women. The present study highlights the link between the onset of falls and reduced affective well-being among men. Avoiding falls may contribute to maintain affective well-being among middle-aged and older men. Further research is required to verify our findings regarding the link between falls and negative affect in women.

Moreover, future longitudinal studies are required in other countries since the link between falls and SWB may vary depending on the cultural background. Additionally, future studies should also explore other potentially mediating and moderating factors such as general self-efficacy (45).

## Data Availability Statement

Publicly available datasets were analyzed in this study. The FDZ-DZA provides access and support to scholars interested in using DEAS for their research. However, for reasons of data protection, signing a data distribution contract is required before data can be obtained. This data can be found at: https://www.dza.de/en/research/fdz/german-ageing-survey/access-to-deas-data.

## Ethics Statement

Ethical review and approval was not required for the study on human participants in accordance with the local legislation and institutional requirements. The patients/participants provided their written informed consent to participate in this study.

## Author Contributions

AH and H-HK contributed to conceptualization and visualization of the study. AH performed data curation, project administration, formal analysis, contributed to methodology, and wrote the first draft of the manuscript. H-HK was responsible for resources and supervision. Both authors contributed to manuscript revision and editing, read, and approved the submitted version.

## Conflict of Interest

The authors declare that the research was conducted in the absence of any commercial or financial relationships that could be construed as a potential conflict of interest.
